# Clinical Midwifery

**Published:** 1849-07

**Authors:** 


*Clinical Midwifery, Comprising the histories of five hundred and forty-five cases of difficult, preternatural, and complicated labours*; with commentaries. By Robert Lee, M. D., F. R. S., etc., etc. First American from the second London Edition. Philadelphia: Lea & Blanchard, 1849, 18 mo. pp, 238.

This is emphatically a book of facts, and deductions. It contains, in a very small compass, a great amount of practical information. The author is well known by his previous contributions to our knowledge of the anatomical composition, and functions of the uterus, and this work will add to his reputation as an useful laborer in the cause of science and humanity.

---

*Mohr, Redwood & Proctor's Pharmacy.* Philadelphia: Lea & Blanchard, 1849.

A multiplicity of authors is hardly to be regarded, prima facie, as any recommendation of a book. But in this volume we do not discover those incongruities and discrepancies, which might be expected in a work, on the title page of which appear the names of three authors, and, to the contents of which, each has largely contributed. The arrangement of the subjects, however, has been materially modified, as well as large additions made by the last named of the trio—the American Author.

The book is strictly practical, and describes only manipulations or methods of performing the numerous processes the Pharmaceutist has to go through, in the preparation and manufacture of medicines, together with all the apparratus and fixtures necessary thereto. On these matters, this work is very full and complete, and details, in a style uncommonly clear and lucid, not only the more complicated and difficult processes, but those not less important ones, the most simple and common. The volume is an octavo of 576 pages. It is elegantly illustrated with a multitude of neat wood engravings, and is unexceptionable in its whole typographical appearance and execution. We take great satisfaction in commending this so much needed treatise, not only to those for whom it is more specially designed, but to the medical profession generally—to every one, who, in his practice, has occasion to prepare, as well as administer remedial agents. For sale by Derby & Co.

* July 18th. Since this article was in type the number of cases have increased to 46 per day. Today the daily report 39 iscases.

